# Clinical evaluation of metagenomic next-generation sequencing for detecting pathogens in bronchoalveolar lavage fluid collected from children with community-acquired pneumonia

**DOI:** 10.3389/fmed.2022.952636

**Published:** 2022-07-15

**Authors:** Wei Guo, Xiaojian Cui, Qiushi Wang, Yupeng Wei, Yanqing Guo, Tongqiang Zhang, Jianghua Zhan

**Affiliations:** ^1^Clinical School of Paediatrics, Tianjin Medical University, Tianjin, China; ^2^Department of Respiratory Medicine, Tianjin Children's Hospital (Tianjin University Children's Hospital), Tianjin, China; ^3^Department of Clinical Lab, Tianjin Children's Hospital (Tianjin University Children's Hospital), Tianjin, China; ^4^Infection Business Unit, Tianjin Novogene Med LAB Co., Ltd., Tianjin, China; ^5^Department of Pediatric Surgery, Tianjin Children's Hospital (Tianjin University Children's Hospital), Tianjin, China

**Keywords:** metagenomic next-generation sequencing (mNGS), bronchoalveolar lavage fluid (BALF), pneumonia, children, pathogen, diagnosis, co-infection

## Abstract

This study is to evaluate the usefulness of pathogen detection using metagenomic next-generation sequencing (mNGS) on bronchoalveolar lavage fluid (BALF) specimens from children with community-acquired pneumonia (CAP). We retrospectively collected BALF specimens from 121 children with CAP at Tianjin Children's Hospital from February 2021 to December 2021. The diagnostic performances of mNGS and conventional tests (CT) (culture and targeted polymerase chain reaction tests) were compared, using composite diagnosis as the reference standard. The results of mNGS and CT were compared based on pathogenic and non-pathogenic organisms. Pathogen profiles and co-infections between the mild CAP and severe CAP groups were also analyzed. The overall positive coincidence rate was 86.78% (105/121) for mNGS and 66.94% (81/121) for CT. The proportion of patients diagnosed using mNGS plus CT increased to 99.18%. Among the patients, 17.36% were confirmed only by mNGS; *Streptococcus pneumoniae* accounted for 52.38% and 23.8% of the patients were co-infected. Moreover, *Bordetella pertussis* and Human bocavirus (HBoV) were detected only using mNGS. *Mycoplasma pneumoniae*, which was identified in 89 (73.55%) of 121 children with CAP, was the most frequent pathogen detected using mNGS. The infection rate of *M. pneumoniae* in the severe CAP group was significantly higher than that in the mild CAP group (*P* = 0.007). The symptoms of single bacterial infections (except for mycoplasma) were milder than those of mycoplasma infections. mNGS identified more bacterial infections when compared to the CT methods and was able to identify co-infections which were initially missed on CT. Additionally, it was able to identify pathogens that were beyond the scope of the CT methods. The mNGS method is a powerful supplement to clinical diagnostic tools in respiratory infections, as it can increase the precision of diagnosis and guide the use of antibiotics.

## Introduction

Community-acquired pneumonia (CAP) is one of the most common and serious childhood infections, threatening the lives and health of children worldwide (1, 2). Timeliness and precision are crucial in the identification of pathogens for guiding targeted antibiotic therapy and predicting prognosis. However, owing to the difficulty in accurately identifying pathogens according to clinical features, some pediatric CAP cases cannot be accurately diagnosed. Bacterial and fungal cultures are the gold standards to identify pathogenic microorganisms, but the culture test's sensitivity is low, time consuming, and unsuitable to identify unculturable bacteria. Virus identification is usually performed by polymerase chain reaction (PCR) and antigen testing, but only specific pathogenic microorganisms can be detected (3, 4). Therefore, these tests were not shown to significantly improve pathogen identification in 21–57% of pediatric patients, even upon application of a combination of these technologies (5–7).

For respiratory tract infections, bronchoalveolar lavage fluid (BALF) is more suitable than sputum for the diagnoses of specific microbiological occurrences and may be less contaminated by oral bacteria during collection (8). Safety and reliability are guaranteed using a safety program to collect BALF (9). Several studies have reported that microbiological examinations of BALF specimens resulted in treatment changes in 38.7–72.7% of infected patients (10, 11).

Metagenomic next-generation sequencing (mNGS) is a culture-independent high-throughput sequencing technology for nucleic acid detection in clinical samples. Unlike conventional tests (CTs), which typically only test for one pathogen, mNGS provides an opportunity to extensively assess pathogens by testing single specimens. The reduced turnaround time makes the mNGS technology more suitable for timely diagnosis. However, mostly respiratory samples are used to study the detection efficiency of mNGS for pneumonia in adults (12, 13), which are not always transferrable to children because of the different composition of respiratory infection pathogens in children and adults. To the best of our knowledge, there have been few studies on the application of mNGS to detect BALF pathogens in children with CAP. Therefore, we conducted a large-scale retrospective trial to assess the clinical significance of mNGS in detecting pathogens in BALF specimens in children with CAP.

## Materials and methods

### Study patients

**Figure 1:**
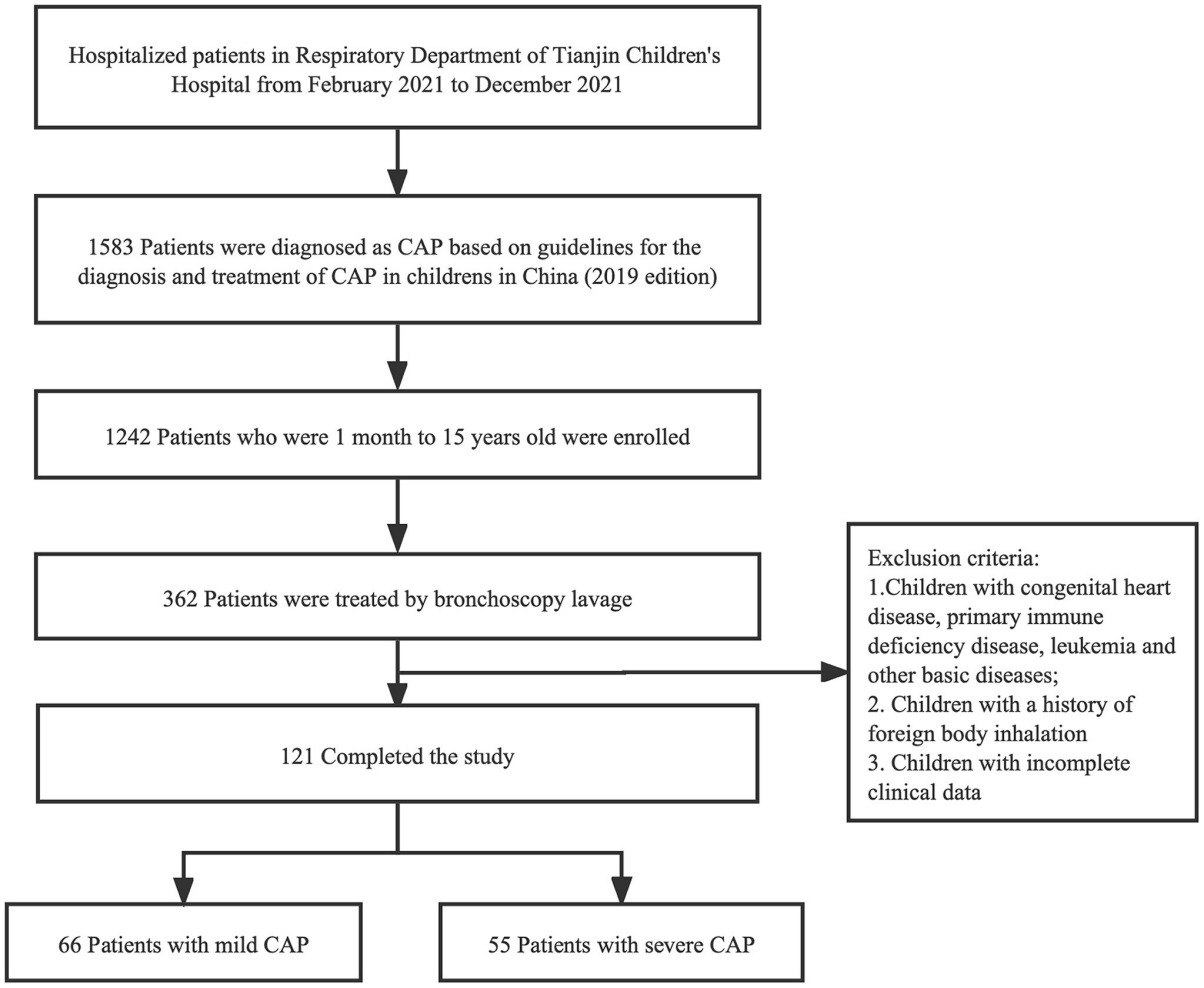
Flowchart of case screening. One hundred twenty-one cases were selected in this study, and categorized into two groups defined as mild CAP group and severe CAP group. CAP, Community-acquired pneumonia.

We retrospectively reviewed 121 cases of CAP at the Respiratory Department of Tianjin Children's Hospital from February 2021 to December 2021 (Figure 1). Tianjin Children's Hospital is a comprehensive pediatric teaching hospital in Tianjin that covers all of North China.

Inclusion criteria were as follows: (i) all patients met the CAP diagnostic criteria (14); (ii) pediatric patients under 15 years of age were selected; and (iii) BALF obtained by bronchoscopy in patients who met the bronchoscopy indications (15). The following conditions will be excluded from the study: (i) presence of any other basic diseases or co-morbidities, including congenital heart disease, primary immune deficiency disease, and leukemia; (ii) a previous history of foreign body inhalation; and (iii) incomplete clinical data. A total of 121 patients were included for further analysis and divided into two groups according to disease severity (Supplementary Table 1). We assigned 66 patients to the mild CAP group and 55 to the severe CAP group. Tianjin Children's Hospital Ethics Committee approved the study (No. L2021-02). This was a retrospective analysis of patient records and patient data was completely anonymized, therefore, the requirement for informed consent was waived.

### Sample acquisition, processing, and CTs

BALF was collected from all patients who underwent bronchoscopy, according to standard procedures. BALF was used for pathogen detection using the mNGS and CT methods. CT methods for BALF included bacterial and fungal cultures as well commercial PCR-based kits for the detection eight common respiratory bacteria (*Streptococcus pneumoniae, Staphylococcus aureus, methicillin-resistant Staphylococcus aureus, Klebsiella pneumoniae, Pseudomonas aeruginosa, Acinetobacter baumannii, Stenostomonas maltophilia*, and *Haemophilus influenzae*) (Capitalbio Jinxin Bio-Technology Co., Ltd., Chengdu, China); *Mycoplasma pneumoniae, Mycobacterium tuberculosis*, Epstein–Barr virus (EBV), cytomegalovirus (CMV), influenza A/B (Da'an Gene Technology Co., Ltd., Guangzhou, China); and adenoviruses (Puruikang Biotech Co., Ltd., Shenzhen, China).

### Laboratory workflow of mNGS

BALF specimens were sent to Tianjin Novogene Medical Laboratory for PD-seq^TM^. DNA was extracted from 300 μL BALF using the TIANMicrobe Magnetic Patho-DNA Kit (NG550, Tiangen Biotech, Beijing, China). DNA libraires were constructed using TIANSeq Fast DNA Library Kit (NG101-02, Tiangen Biotech, Beijing, China). DNA fragments of size 150–200 bp were obtained using the enzyme, followed by terminal repair, phosphorylation, and A-tailing reactions. Illumina adapters were ligated onto the A-tailed fragments. Fragments with adapters were purified and amplified using PCR. After purifying the PCR product, the libraries were pooled and sequenced for 50 bp single ends or 75 bp single ends on an Illumina NovaSeq machine. The data output of each sample was guaranteed to be more than 20 M reads. Nuclease free water and plasma from a non-infected individual were used as negative controls to monitor contamination throughout the workflow.

### Bioinformatic analyses and interpretation

Adapters and the reads of low quality or length < 15 bp were filtered out using fastp (16). Bowtie2 (version 2.3.5.1) (17) was used to map human reference genomes [GRCh38+YHref (18)] to identify and remove the human sequencing data. After removing human reads, the remaining reads were aligned with our curated microbial genome database (PD-seq^TM^ database version 1.0), whose genome sequences were derived from the RefSeq, GenBank, and NT databases. The database used in this study contained 9,295 bacterial species, 7,210 viral species, 412 fungal species, 104 parasites. We identified the species by mapping our curated microbial database using the Kraken2+Bracken workflow. Kraken2 (version2.1.2) (19) was used to annotate the sequence reads. Species abundance was estimated using the Bracken software (version 2.6) (20). The determination of true positive results was carried out as follows: (i) the specific sequence number of clinical reportable pathogens (CRP) or non-CRP in the patient sample should have been more than 3 times the corresponding reading in the negative control, and the relative abundance of non-CRP was more than 10%; (ii) the specific sequence number of *M. tuberculosis* in the patient sample was more than 1; (iii) the true positive species detected in the same batch should have been 1/1,000 or more of strong positive (more than 1,000 specific sequences), considering the influences caused by “cross-contamination” or “spillage”; (iv) it was efficient that more than 200 reads of the internal control (Spike-in Control I) (Zymo Research, Irvine, California, USA) spiked in negative control and all samples were detected; (v) species detected in negative controls with a frequency of more than 25% were regarded as “environmental pollutants” and would be removed; (vi) species colonizing the respiratory tract were filtered into the microecology in the final report. For suspected positive pathogens that were initially screened using the above steps, BLAST verification was performed. For positive pathogens, samtools (21) was used to get coverage and average depth.

### Clinical composite diagnosis

Pathogen diagnosis required comprehensive judgment of clinical symptoms, laboratory examination, imaging and microbiological test (including nucleic acid detection of respiratory pathogens, BALF culture, and mNGS), and treatment response. Clinicians, laboratory physicians, and experienced mNGS report interpretation experts participated in the final decision after an in-depth discussion.

### Statistical analysis

The mean ± standard deviation (SD) was used to represent numerical variables with normal distributions. Percentages were used to represent categorical data. Chi-square test and student's *t*-test were used for comparative analysis of categorical variables and numerical variables, respectively. *P* < 0.05 was considered statistically significant.

## Results

### Clinical features of patients

Our study included 66 patients in the mild CAP group and 55 in the severe CAP group. Table 1 and Supplementary Table 2 show the clinical characteristics of the two groups. The age of patients in the severe CAP group was significantly higher than that of those in the mild CAP group. *M. pneumoniae* is the main pathogen causing respiratory infections in school-age children and adolescents with severe clinical symptoms (22). Unexpectedly, patients in the severe CAP group had a longer mean hospital stay than those in the mild CAP group (*P* = 0.001). The neutrophil rate in the severe CAP group was significantly higher than that in the mild CAP group (*P* = 0.022), and the lymphocyte ratio was significantly lower than that in the mild CAP group (*P* = 0.029). A higher neutrophil proportion indicates more severe bacterial and/or mycoplasma infection (23). However, there was no significant difference in white blood cell count, C-reactive protein, and procalcitonin levels between the two groups. The number of patients with hemoptysis and/or blood in the phlegm in the severe CAP group was significantly higher than that in the mild CAP group (*P* = 0.028), while there was no significant difference in other symptoms (such as non-productive cough, productive cough, thoracalgia, etc.) and underlying pulmonary diseases. The imaging results (except for “Consolidations in multiple lobes” and “Pulmonary interstitial lesions”) in the two groups were significantly different, suggesting that imaging features can indicate the disease's severity.

**Table 1:** Comparison of clinical features of patients with mild and severe community-acquired pneumonia.

**Clinical characteristics**	**Mild CAP group** **(*****n*** = **66)**	**Severe CAP group** **(n** = **55)**	***P*** **value**
**Characteristics**			
Age, years (mean ± sd)	4.44 ± 2.77	5.64 ± 2.85	0.020
Sex, female, *n* (%)	31 (46.97%)	29 (52.73%)	0.528
Hospital, days (mean ± sd)	5.77 ± 1.95	8.25 ± 2.95	0.000
**Laboratory parameters**			
White blood cell counts (10^9^/L) (mean ± sd)	10.14 ± 4.05	9.57 ± 5.98	0.536
Neutrophil [%] (mean ± sd)	57.81 ± 17.65	64.09 ± 10.37	0.022
Lymphocyte [%] (mean ± sd)	32.14 ± 15.05	26.93 ± 9.73	0.029
C-reactive protein (mg/L) (mean ± sd)	27.74 ± 46.31	23.35 ± 22.81	0.523
Procalcitonin (ng/mL) (mean ± sd)	0.84 ± 2.92	0.39 ± 0.89	0.268
**Presenting symptoms or signs**			
Non-productive cough, *n* (%)	3 (4.55%)	4 (7.27%)	0.522
Productive cough, *n* (%)	62 (93.94%)	50 (90.91%)	0.527
Thoracalgia, *n* (%)	1 (1.52%)	1 (1.82%)	0.896
Chest tightness and/or wheezing, *n* (%)	9 (13.64%)	9 (16.36%)	0.675
Hemoptysis and/or blood in phlegm, *n* (%)	1 (1.52%)	6 (10.91%)	0.028
Fever, *n* (%)	62 (93.94%)	54 (98.18%)	0.243
Fatigue, *n* (%)	3 (4.55%)	11 (20%)	0.008
**Underlying pulmonary disease**			
Bronchial asthma, *n* (%)	1 (1.52%)	1 (1.82%)	0.896
Bronchiectasis, *n* (%)	2 (3.03%)	0 (0%)	0.193
Previous history of tuberculosis, *n* (%)	3 (4.55%)	0 (0%)	0.109
**Imaging features**			
Consolidations in multiple lobes, *n* (%)	32 (48.48%)	26 (47.27%)	0.894
The range of pulmonary inflammatory consolidation is >1/2, *n* (%)	8 (12.12%)	29 (52.73%)	0.000
Bronchopatency, *n* (%)	26 (39.39%)	36 (65.45%)	0.004
Atelectasis, *n* (%)	7 (10.61%)	21 (38.18%)	0.000
Pleural effusion, *n* (%)	1 (1.52%)	12 (21.82%)	0.000
Pulmonary interstitial lesions, *n* (%)	2 (3.03%)	2 (3.6%)	0.853

*CAP, Community-acquired pneumonia*.

### Comparison of the diagnostic performances of mNGS and CT methods

**Figure 2:**
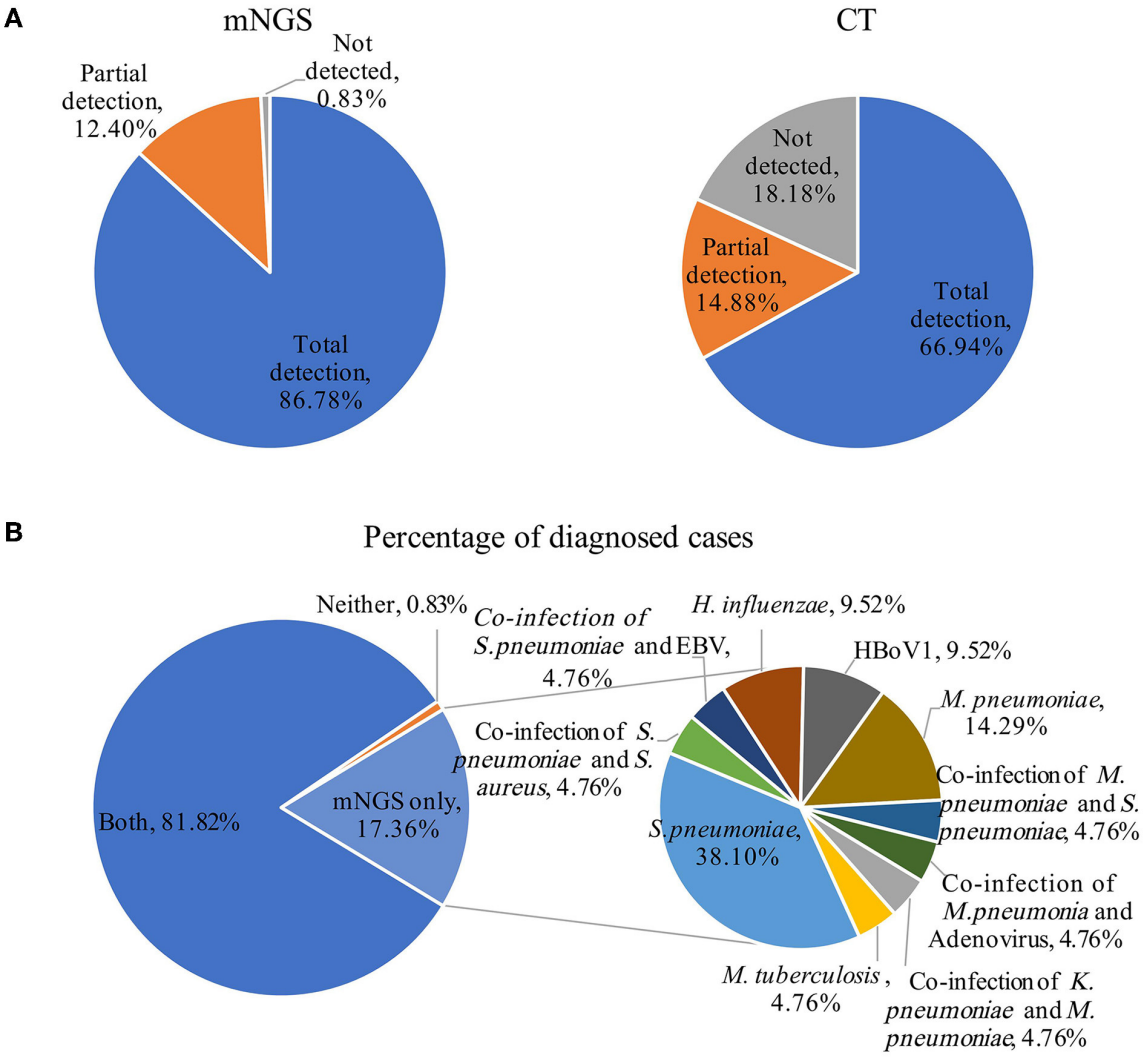
Comparison of pathogens detection using the metagenomic next generation sequencing (mNGS) and conventional tests (CT) methods for the diagnosis of community-acquired pneumonia. **(A)** Using the final clinical diagnosis as references, the percentages of patients with complete detection, partial detection, and no pathogen detection using the mNGS and CT method. **(B)** The impact of mNGS in pathogen diagnosis, percentage of patients were diagnosed using both the mNGS and CT methods, neither and mNGS only (pie chart on the left), pathogens identified only using mNGS contribute to diagnosis (pie chart on the right). EBV, Epstein–Barr virus.

Using the final clinical diagnosis as references, we evaluated performances of mNGS and CT methods for pathogen identification. The etiological diagnosis results for all patients are listed in Supplementary Table 3. Overall, the positive coincidence rate of the mNGS method was 86.78% (105/121), whereas that of the CT method was 66.94% (81/121). Thus, the positive coincidence rate of the mNGS method was much higher than that of the CT method (*P* < 0.001). The mNGS method partially identified pathogens in 15 of the 121 cases and no pathogens in one case. However, the CT method partially identified pathogens in 18 of the 121 cases and could not identify any pathogens in 22 of the 121 cases (Figure 2A). Although all diagnoses partially depended on the clinical manifestations, the final pathogenic diagnoses were 19 cases (17.36%) and 99 cases (81.82%) using only mNGS and both mNGS and CT methods, respectively (Figure 2B). For one case in which both the mNGS and CT methods were negative, the clinical data showed that it was endo-bronchial tuberculosis. Bacteria and viruses that caused pneumonia and were identified only by mNGS included *S. pneumoniae* (*n* = 8), *M. pneumoniae* (*n* = 3), *H. influenzae* (*n* = 2), *HBoV1* (*n* = 2), and *M. tuberculosis* (*n* = 1). In addition, two organisms (*n* = 5) per sample identified only by mNGS included co-infection of *K. pneumoniae* and *M. pneumoniae, S. pneumoniae* and *S. aureus, S. pneumoniae* and EBV, *M. pneumoniae* and *S. pneumoniae, M. pneumoniae* and *Adenovirus*. The possible reasons for the missed detection by CT methods were that the bacterial load was lower than the limit of detection of the PCR assays, or viruses (*HBoV1*) that were not included in the CT tests. These bacterial-bacterial and bacterial-viral co-detections indicate the complex etiologies of some CAP patients and demonstrate the capability of mNGS to detect all pathogens in a single experiment.

### Comparison of pathogens detected using the mNGS and CT methods

**Figure 3:**
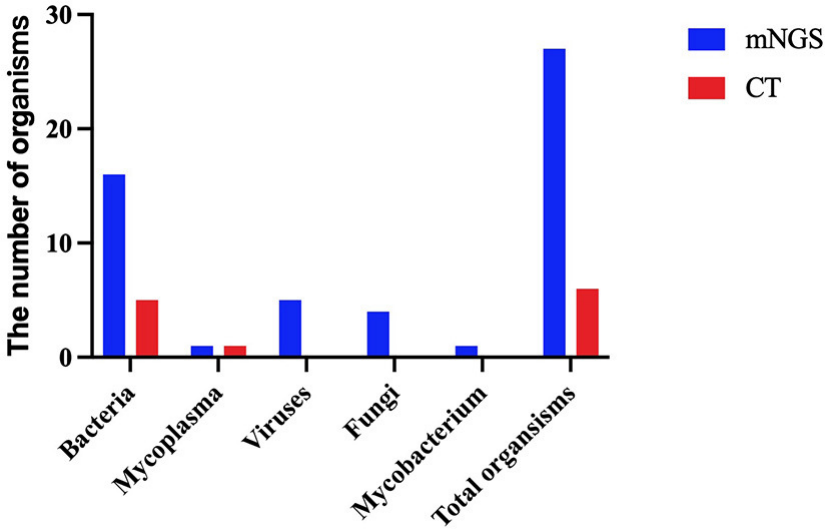
A comparison of the type of organisms detected using the metagenomic next-generation sequencing (mNGS) and conventional tests (CT) methods.

**Figure 4:**
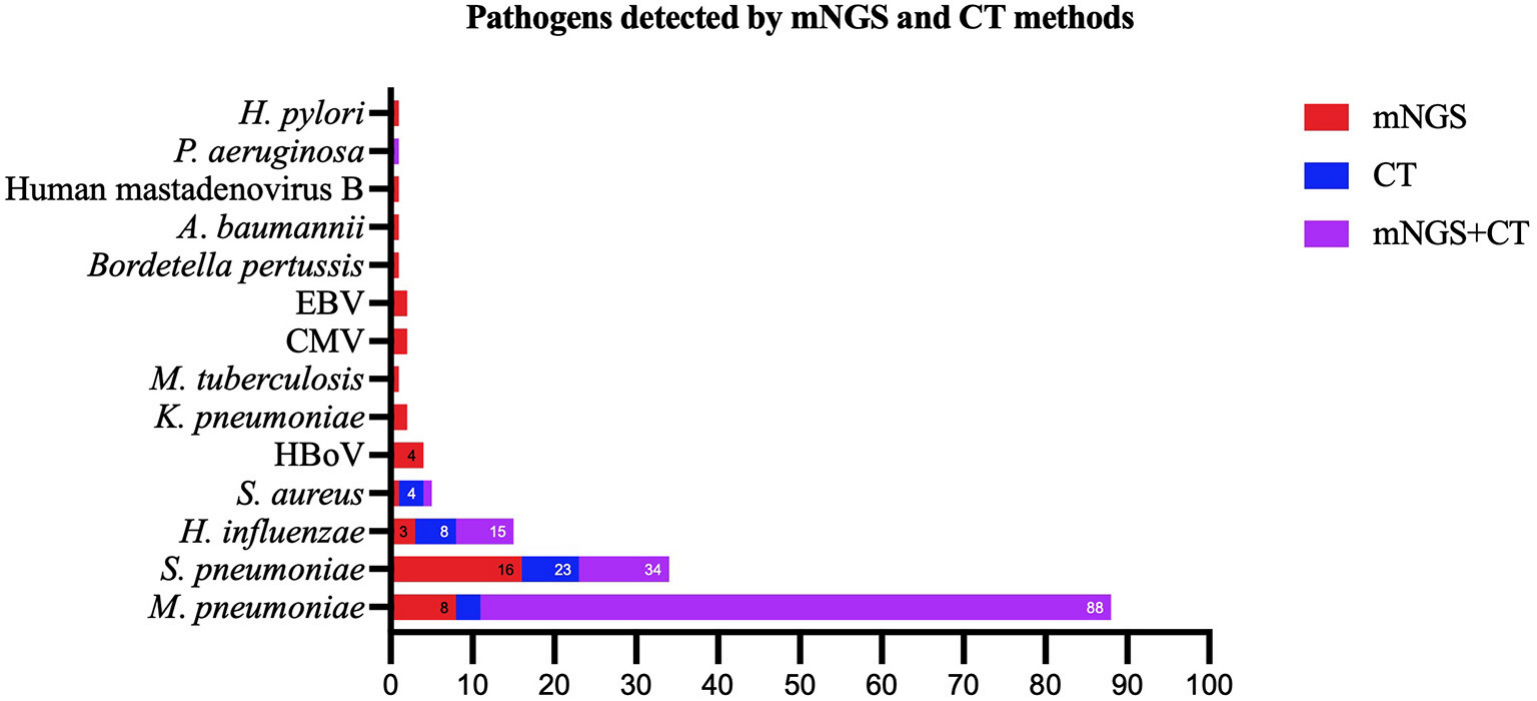
Pathogens identified using both the metagenomic next generation sequencing (mNGS) and conventional tests (CT) methods, mNGS only, and CT methods only. mNGS, metagenomic next generation sequencing; CT, conventional test, HBoV, Human bocavirus; CMV, Cytomegalovirus; EBV, Epstein–Barr virus.

**Figure 5:**
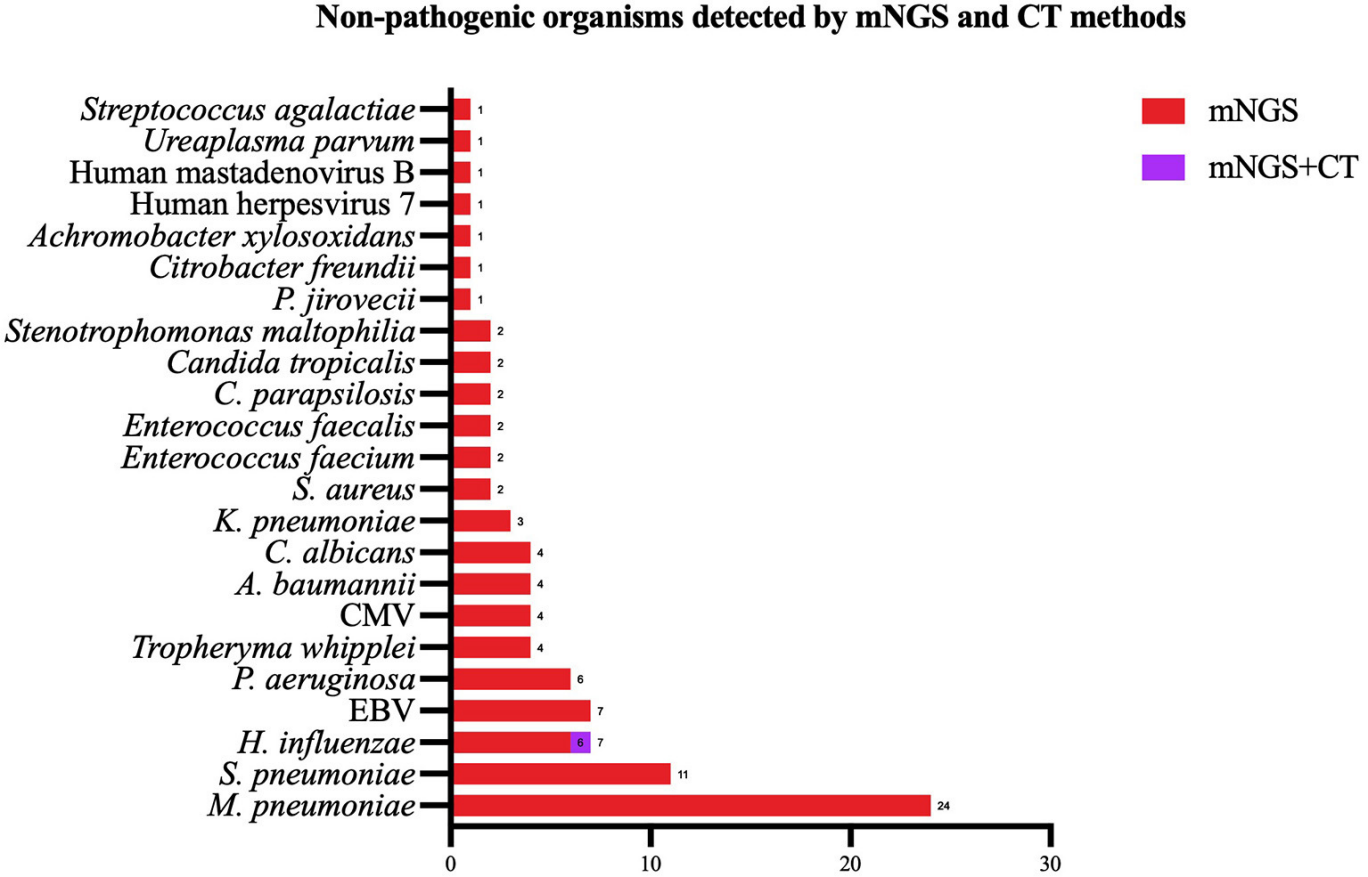
Non-pathogenic pathogens identified using both the metagenomic next generation sequencing (mNGS) and conventional tests (CT) methods, mNGS only. mNGS, metagenomic next generation sequencing; CT, conventional test, HBoV, Human bocavirus; CMV, Cytomegalovirus; EBV, Epstein–Barr virus.

Except for common colonizing organisms or contaminating organisms, a total of 27 organisms were identified using mNGS, whereas six were identified using the CT method. mNGS identified more bacterial (16 vs. 5), viral (5 vs. 0), fungal (4 vs. 0), and mycobacterial (1 vs. 0) organisms than the CT method (Figure 3). *Mycoplasma* was detected using both methods. The top three pathogens in 121 CAP patients were *M. pneumoniae* (88/121), *S. pneumoniae* (34/121), and *H. influenzae* (15/121). Except for that of *H. influenzae* and *S. aureus*, the positivity rate of mNGS for pathogenic bacteria was higher than that of the CT method. Additionally, *K. pneumoniae, M. tuberculosis, B. pertussis, A. baumannii, P. aeruginosa*, and *Helicobacter pylori* were detected using mNGS alone, and the CT results of these microorganisms were all negative. Viruses could only be detected using mNGS. HBoV, EBV, CMV, and Human mastadenovirus B were considered pathogenic according to the reads and relatively high abundance and clinical manifestations of the patients (Figure 4). Additionally, other pathogens which were considered to be non-pathogenic were also detected (Figure 5). *Candida albicans, C. parapsilosis, C. tropicalis*, and *Pneumocystis jirovecii* detected only by mNGS were considered to be non-pathogenic because the patients involved were not immunocompromised and did not use large amounts of glucocorticoids in the past. However, among the non-pathogenic organisms, pathogens such as *M. pneumoniae, H. influenzae*, and *S. aureus* were “usually” considered to cause CAP. As there were no detailed uniform criteria or authoritative guidelines for interpreting mNGS reports, we determined whether the pathogens were pathogenic, colonizing, or contaminants based on clinical expertise, patient imaging findings, and inflammation indicators.

### Differences in the pathogen spectrum between the mild CAP and severe CAP groups

**Figure 6:**
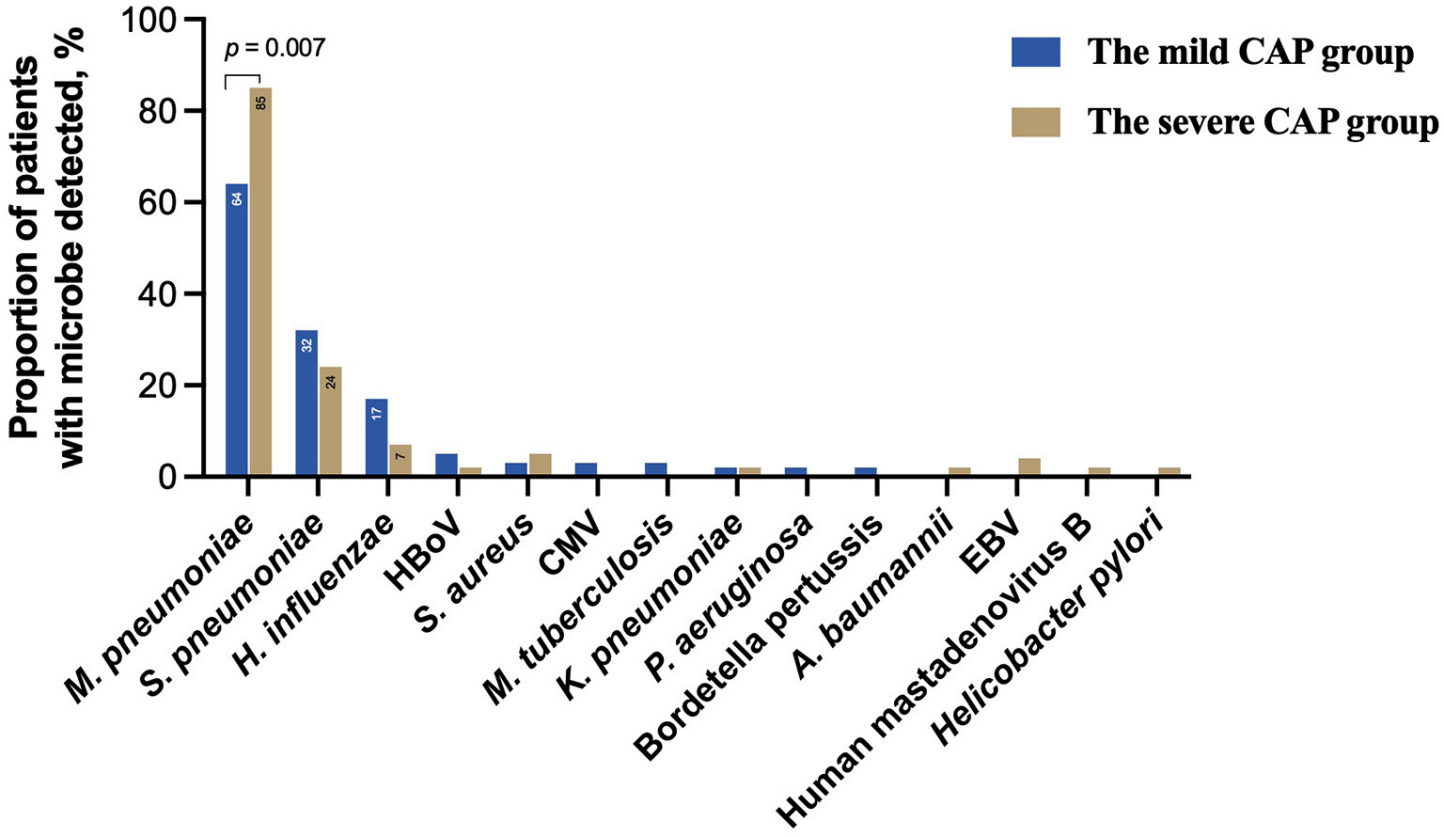
Clinically determined pathogens spectrum between the mild CAP and severe CAP groups. CAP, community-acquired pneumonia; HBoV, Human bocavirus; CMV, Cytomegalovirus; EBV, Epstein–Barr virus.

**Figure 7:**
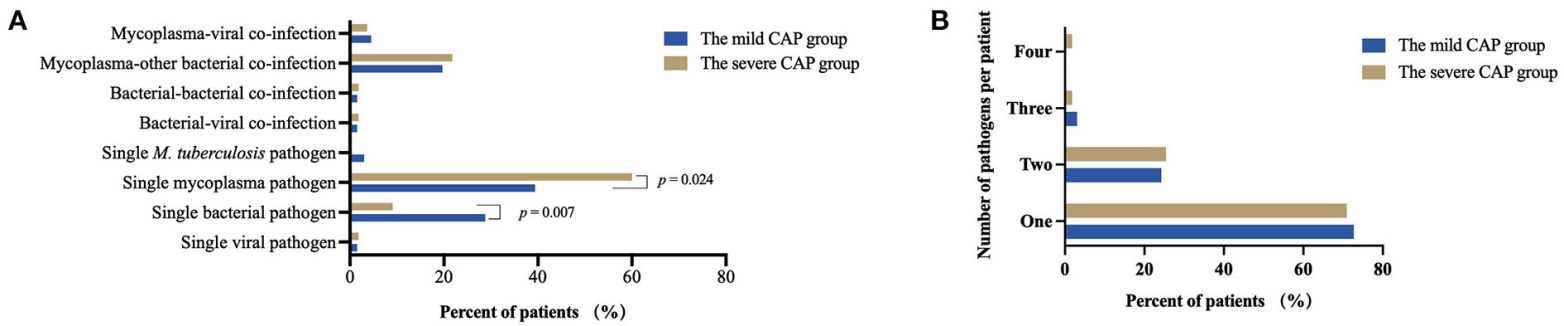
Common combinations of single pathogen infection and co-infection **(A)** and total number of pathogens per patient **(B)** between the mild CAP and severe CAP groups. CAP, community-acquired pneumonia.

To further evaluate the microbial etiology in children with CAP, the distribution of pathogens in the mild and severe CAP groups was analyzed in our study. Differences in pathogen profiles between the two groups were shown in Figure 6. The infection rate of *M. pneumoniae* in the severe group was significantly higher than that in the mild group (*P* = 0.007). CMV, *M. tuberculosis, P. aeruginosa*, and *B. pertussis* were detected in the mild CAP group but not in the severe CAP group. *A. baumannii*, EBV, Human mastadenovirus B, and *H. pylori* were detected only in the severe CAP group. When grouped by combinations of single-pathogen infection and co-infection, the severe CAP group showed a significantly higher percentage of single mycoplasma pathogens than the mild CAP group (*P* = 0.024) (Figure 7A). Additionally, single bacterial pathogen (*Mycoplasma* was not included) infections were significantly overrepresented in the mild CAP group. This suggests that the symptoms of other single bacterial infections are milder than those of mycoplasma infections in the region. However, there was no significant difference in co-infection between the mild and severe cases. Although two or more pathogens are prevalent in some CAP cases, a causal relationship between the number of pathogens and the severity of the disease has not been found (Figure 7B).

## Discussion

In this study, 27 organisms were detected using mNGS in 121 BALF specimens, among which the top three pathogens were *M. pneumoniae, S. pneumoniae*, and *H. influenzae*, which are common pathogenic bacteria in children with CAP. Wang et al. (24) obtained similar results by detecting the pathogenic spectrum of children with severe non-responding pneumonia using mNGS. In contrast, Cui et al. (25) reported *M. pneumoniae*, respiratory syncytial virus, *K. pneumoniae*, and *S. pneumonia*e as the leading causes of lower respiratory tract infections among children with severe pneumonia in southern China, based on mNGS performed on BALF samples. Different pathogenic spectra may be related to the different ages and regions of the children. The children in the study by Cui et al. were 1–6 years old with an average age of 3.3 ± 1.8 years old, which was significantly younger than the age group of children in our study (1 month−15 years old with an average age of 5.6 ± 3.1 years old). Zhan et al. (26) detected pathogens in 57 immunocompetent adults with pneumonia using mNGS, and their results showed that *M. pneumoniae, C. albicans*, Influenza A virus, and adenovirus were the main pathogens, suggesting that there were differences between the common pathogens of pneumonia in adults and children. However, based on the above analysis, it can be concluded that *M. pneumoniae* is an important cause of pneumonia in all ages.

Our study used a comprehensive clinical diagnosis as the reference standard, and the positive coincidence rate of mNGS was significantly higher than that of CTs (*P* < 0.001). Most of the microorganisms missed by CTs were bacteria. Although the culture method is the gold standard, the positive rate of the culture method in diagnosing lower respiratory tract infections in children is very low (27). Taking *S. pneumoniae* as an example, the positive rate of the culture method in this study was only 8.82%. If the culture method is used as the gold standard, many children who tested positive by mNGS would have been identified as false positives, resulting in a low specificity of the mNGS method. Therefore, there are deviations in the evaluation of the sensitivity, specificity, positive predictive value, and negative predictive value of the mNGS method. For a more intuitive comparison, our study calculated the detection rates of the two methods for various pathogens instead of using sensitivity and specificity. The results showed that the pathogen detection rates of the mNGS method were higher than the culture method, especially for *S. pneumoniae* and *M. pneumoniae*.

Compared to CTs, mNGS can identify more organisms, especially fastidious and atypical organisms that cause pneumonia (28). In this study, *B. pertussis, H. pylori, A. baumannii, K. pneumoniae*, and *M. tuberculosis* were diagnosed as pathogens detected only by mNGS but were negative by CT. *B. pertussis* and *H. pylori* are usually not considered by clinicians in CTs for patients with pneumonia if there are no obvious clinical features or strong suspicions of infections. *A. baumannii* is a non-fermentative bacterium that is difficult to culture because of its unique culture conditions. Although there is a PCR test available, the low bacterial load in the specimen was below the minimum detection limit of 5 × 10^2^ copies in this study. However, *A. baumannii* was detected in five patients using mNGS, which may be attributed to the higher sensitivity of the test. *K. pneumoniae* and *A. baumannii* are conditional pathogens; therefore, clinical symptoms and mNGS results (including but not limited to microbial sequence number and relative abundance) need to be considered by clinicians to determine if they are pathogenic. In our study, they were identified as pathogenic in 3 of the 10 cases. Among the two tuberculosis cases, one case was positive and one case was negative on mNGS. The negative case may have been due to low bacterial load beyond the limit of detection of mNGS or due to an inefficient DNA extraction procedure, thus leading to a missed detection. Thus, mNGS could be a useful supplement in clinical practice for detecting *M. tuberculosis*.

The apparent advantage of mNGS in virus detection has been gradually reported in recent years (29, 30), especially after the successful detection of SARS-Cov-2 using mNGS technology (31). In this study, 4 cases were identified as HBoV infections. Like *B. pertussis* and *H. pylori*, HBoV was not included in our conventional clinical tests because of the lack of PCR kits. HBoV is a parvovirus that mainly affects infants but can also infect adults. Single infections with HBoV are rare, while co-infections with other respiratory pathogens are frequently observed. In China, the prevalence rates of HBoV infection and co-infections were 5 and 50.3%, respectively, from 2005 to 2016 (32). Similarly, 2 of 4 (50%) cases of HBoV infection in this study were co-infected with other respiratory bacteria. This further highlights that mNGS has advantages in the detection of pathogen co-infections, which can be missed by conventional tests.

A previous study showed that mNGS was more sensitive than CTs in diagnosing mixed infections in the lung (33). In our study, 34 cases were diagnosed with co-infections. Among them, only 12 (35.29%) were positive in conventional tests, while 19 (55.88%) were mNGS-positive. Co-infections in 5 cases were detected using mNGS alone. A total of 11 cases were diagnosed with *M. pneumoniae* and *S. pneumoniae* co-infections, which was the most frequent co-infection combination (see Supplementary Table 3). Compared with PCR kits targeting several microorganisms, mNGS can have a broader spectrum of pathogens and a lower detection limit. These are considered advantages for identifying mixed infections. At the same time, mNGS provides a pathogenic basis for changing the anti-infection treatment regimen and shortens the detection time of multiple pathogens so that infected patients can receive timely treatment.

mNGS contributes to accurate diagnosis and rational antibiotic treatment of culture-negative infections (34). In this study, some pathogens detected using mNGS were negative on CT. *A. baumannii* is a non-fermentative bacterium that is insensitive to conventional broad-spectrum antibiotics. If pathogens are detected by mNGS in time, antibiotics can be adjusted to include drugs containing enzyme inhibitors, which are crucial for treating children with CAP (35). HBoV can cause mild and severe respiratory tract infections in children (36). The clinical manifestations of severe HBoV infection are often atypical, including fever, cough, wheezing, and dyspnea. The early administration of glucocorticoids and human immunoglobulins is essential for controlling disease progression. Our study found four cases of HBoV infection, which could easily be misdiagnosed as bacterial-viral co-infection without mNGS detection. Pertussis is an acute respiratory infection caused by *B. pertussis*. Most infants do not have typical clinical manifestations, and most older children are misdiagnosed with a chronic cough or allergic diseases (37). In this study, mNGS was used to diagnose a child with *B. pertussis* infection, and timely treatment with azithromycin effectively blocked disease transmission. *K. pneumoniae* is a common pathogen of hospital-acquired and community-acquired infections that can lead to severe pneumonia, urinary tract infections, and bloodstream infections with a high mortality (38). Early use of effective antibiotics is key for successful patient management. In this study, we found two *K. pneumoniae* cases, suggesting that mNGS should be used in time to identify pathogenic bacteria in patients with pneumonia and to guide antibiotic use.

mNGS is characterized by the objective detection of known and unknown pathogens in affected individuals. Unlike CTs, which target certain pathogens, mNGS covers all microorganisms. Therefore, the exclusion of colonizing species and environmental organisms is key to screening pathogens from a multitude of microorganisms detected using mNGS in clinical specimens. Zhan et al. (26) evaluated the clinical guidance of PneumoSeq for microbial detection based on the classification of microorganisms, including common pathogens of pneumonia, atypical pathogens, special bacteria, respiratory microecological bacteria, fungi, respiratory viruses, and microorganisms with unclear significance. Peng et al. (30) defined clinically significant microorganisms (CSMs) with a relative abundance >30% at the species level and in the literature supporting their pathogenicity. Additionally, mycobacteria and molds were considered CSMs when the sequence numbers at the species level were >3 and 10, respectively. Oral commensals were not considered as CSMs. In summary, various criteria have been reported by different studies when interpreting mNGS results. In our study, environmental contaminants and colonizing species determined by professional interpreters and clinicians were excluded based on certain screening principles (as described in Materials and Methods). It has been further proven that this study's value is in providing a data reference for the formulation of interpretation standards.

This study has several limitations. First, considering the population characteristics, an infrequent RNA virus infection in children with CAP, and the high cost of mNGS, RNA sequencing was not performed in this study, which may have resulted in missing several RNA viruses (such as respiratory syncytial virus and rhinovirus). Second, interpreting mNGS reports relies on experienced interpreters, and there is a lack of standard criteria. As with all new technologies, there are still significant difficulties and challenges in interpreting and reporting the data. Therefore, there is a high variability between laboratories in identifying microorganisms and true pathogens (39). This is related not only to different laboratory environments and sequencing platforms but also to the expertise and experience of interpreters. Third, a limited sample size and all patients enrolled from a single hospital may lead to biased conclusions. Finally, because the data were retrospective, mNGS was performed after the patients had already been treated; therefore, the therapeutic effect of mNGS could not be evaluated.

## Conclusion

The positive coincidence rate of the mNGS method for pathogens was much higher than that of the CT method (*P* < 0.001) in children with CAP. The mNGS method is an effective supplement for pathogen diagnosis in children with CAP, as CT methods combined with mNGS could confirm the diagnosis in 99.17% of the patients. Thus, mNGS should be performed on CT-negative specimens to guide rational clinical drug use and further reduce antibiotic abuse.

## Data availability statement

The raw sequence data reported in this paper have been deposited in the Genome Sequence Archive (Genomics, Proteomics and Bioinformatics 2021) in National Genomics Data Center (Nucleic Acids Res 2022), China National Center for Bioinformation / Beijing Institute of Genomics, Chinese Academy of Sciences (GSA: CRA006760) that are publicly accessible at https://ngdc.cncb.ac.cn/gsa.

## Ethics statement

The studies involving human participants were reviewed and approved by Tianjin Children's Hospital Ethics Committee. Written informed consent from the participants' legal guardian/next of kin was not required to participate in this study in accordance with the national legislation and the institutional requirements.

## Author contributions

The study was supervised by YG, TZ, and JZ. The work was conceived and the manuscript was written by QW, XC, and WG. Clinical data collection and sample collection were done by YW, XC, and TZ. Statistical analyses and manuscript editing were done by QW. All authors contributed to the article and approved the submitted version.

## Funding

This research was supported by National Natural Science Foundation of China (Grant Number 81771589), the General Program of Tianjin Natural Science Foundation (Grant Number 21JCYBJC00460), and the Program of Tianjin Science and Technology Talent Cultivation (Grant Number RC20020).

## Conflict of interest

QW and YG are employed by Tianjin Novogene Med LAB Co., Ltd., (Tianjin, China). The remaining authors declare that the research was conducted in the absence of any commercial or financial relationships that could be construed as a potential conflict of interest.

## Publisher's note

All claims expressed in this article are solely those of the authors and do not necessarily represent those of their affiliated organizations, or those of the publisher, the editors and the reviewers. Any product that may be evaluated in this article, or claim that may be made by its manufacturer, is not guaranteed or endorsed by the publisher.
